# Fulminant hemophagocytic lymphohistiocytosis induced by pandemic A (H1N1) influenza: a case report

**DOI:** 10.1186/1752-1947-5-280

**Published:** 2011-07-03

**Authors:** Christophe Willekens, Aurélie Cornelius, Mary-Jane Guerry, Agnès Wacrenier, François Fourrier

**Affiliations:** 1 Service de Réanimation Polyvalente, Hôpital Roger Salengro, Rue Emile Laine, CHRU, Lille, France; 2 Service des Maladies du Sang, Hôpital Huriez, Lille, France; 3 Pôle de Pathologie-Anatomie et Cytologie Pathologiques, Centre de Biologie-Pathologie, Lille, France

## Abstract

**Introduction:**

Hemophagocytic lymphohistiocytosis induced by viral diseases is a well recognized entity. Severe forms of H5N1 influenza are known to be associated with symptoms very similar to a reactive hemophagocytic syndrome. We report a case of fulminant lymphohistiocytosis associated with the pandemic A (H1N1) variant.

**Case presentation:**

A 42-year-old Caucasian woman developed a syndrome of fatal hemophagocytic lymphohistiocytosis shortly after H1N1 influenza. Initial symptoms of the viral disease were unusual, with acute abdominal involvement. Our patient's course was complicated by diffuse skin rash and ileal ischemia. Our patient died of refractory shock and multi-organ failure. Skin, ileum and colon histology was consistent with an acute apoptosis combined with an increased cellular regeneration.

**Conclusions:**

Influenza may be complicated by severe forms of hemophagocytic lymphohistiocytosis. To ensure early recognition and treatment, physicians should be aware of the possible induction of the syndrome by the novel H1N1 variant. The rapid occurrence of a multi-organ involvement with evocative biological features of macrophage activation should alert clinicians.

## Introduction

Virus-associated hemophagocytic lymphohistiocytosis (HLH) is a well recognized entity [1]. Most cases are related to Epstein-Barr virus (EBV), cytomegalovirus (CMV), and herpes virus infections. Influenza-induced macrophage activation syndrome is very rare. It has been described in isolated case reports of immunocompromised or immunocompetent patients suffering from either avian or swine influenza. Consistent with the deregulation of macrophage function by influenza virus, severe forms of H5N1 influenza are known to be associated with symptoms very close to a reactive hemophagocytic syndrome [2]. Moreover, recent autopsy findings of patients suffering from H1N1 pandemic influenza have shown a high proportion of hemophagocytosis in bone marrow, lymph nodes, and spleen [3]. We report a case of fulminant HLH related to H1N1 pandemic influenza.

## Case presentation

A 42-year-old Caucasian woman was admitted to the emergency ward of our hospital with severe gastroenteritis. On admission, she was febrile (38.2°C), dehydrated, and oliguric. Laboratory results were consistent with severe extracellular dehydration, increased inflammation markers, acute renal failure, and abnormal liver function tests. Leukocyte and platelet counts were 33 × 10^3 ^and 274 × 10^3 ^cells/mL, respectively. An abdominal computed tomography (CT) scan showed a diffuse ileocolitis. Despite volume repletion and antibiotics, our patient continued to deteriorate. She was transferred to our intensive care unit (ICU) with cardiorespiratory, renal, and hepatic failure. In the subsequent 48 hours, she developed diffuse cutaneous erythema, leukopenia (0.72 × 10^3 ^cells/mL), and thrombocytopenia (22 × 10^3 ^cells/mL), associated with very high levels of lactate dehydrogenase (LDH) (9,500 IU; n<240), tryglycerides (11.9 g/L), and ferritin (11,060 ng/mL). A whole body CT scan revealed signs of mesenteric ischemia with hepatomegaly (19 cm) and a heterogeneous spleen. HLH was suspected because of her persistent fever, severe pancytopenia, hyperferritinemia, hypertriglyceridemia, increased LDH and hepatomegaly. A bone marrow biopsy was performed and the results revealed typical hemophagocytosis.

No family history of HLH was found and a primary familial form was considered unlikely. According to the CT scan and bone marrow biopsy results, no hematological malignancy was thought to be responsible for a secondary form. Blood protein electrophoresis showed hypo-γ-globulinemia (7.5 g/L) with a low IgG level (5.5 g/L). All autoimmune antibody test results were negative. Despite an extensive multi-site sampling and serological studies, no bacterial or fungal infection was found. Laboratory tests for viral infection including mumps, measles, EBV, CMV, herpes simplex virus (HSV), human herpesvirus 6 (HHV6), HHV8, varicella zoster virus (VZV), B19 parvovirus, adenovirus, enteroviruses, viral hepatitis B and C, HIV, Hantaan virus, and human T cell lymphotropic virus (HTLV) were unremarkable. Only nasopharyngeal polymerase chain reaction (PCR) testing was positive for influenza A (H1N1).

On day four after admission to the ICU, in consideration of our inability to exclude a malignant lymphoma with certainty, corticosteroid therapy and rituximab (375 mg/m^2^) were initiated. Due to persistence of shock and respiratory failure, our patient was considered unable to receive etoposide. An exploratory laparotomy showed a mesenteric ischemia extending to the ileum and right colon. Despite ileum resection, circulatory support, high flow hemofiltration, and mechanical ventilation, our patient died seven days after admission with multi-organ failure and refractory circulatory shock. The positive results of the PCR were obtained after her death, and our patient did not receive antiviral therapy.

**Figure 1:**
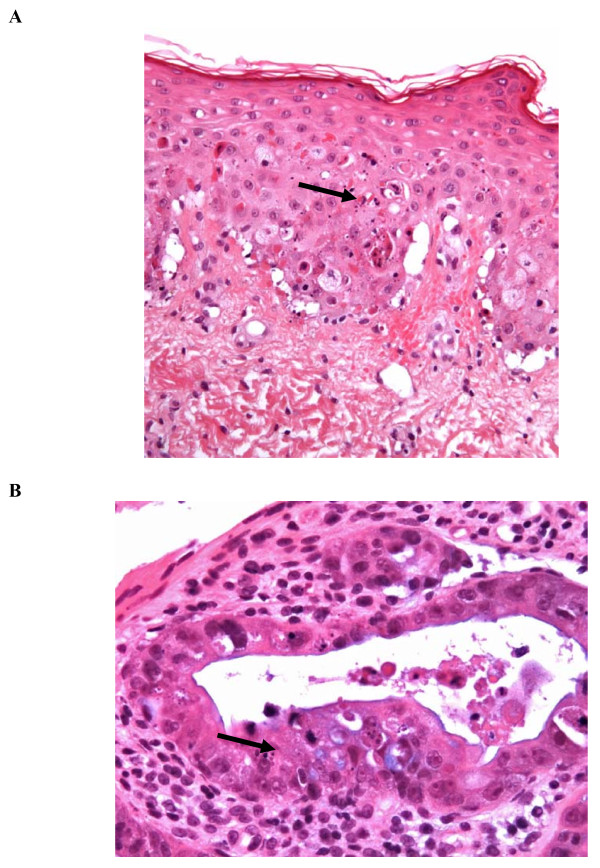
**Photography of skin and colon microscopic pathology**. A) Skin biopsy (hematoxylin and eosin stain, ×200): an important epidermal apoptosis (black arrow) associated with basal cell hydropic degeneration can be seen in the epidermis. B) Colon: (hematoxylin and eosin stain, ×400): an increased number of apoptotic bodies combined with an increased cellular regeneration can be seen; crypt proliferative zones include many apoptotic bodies (black arrow).

A skin biopsy, and ileum and colon histology were examined for pathological changes. Skin biopsy results revealed major epidermal apoptosis with basal cell hydropic degeneration and sub-epidermal blistering. A moderate and predominantly peri-vascular infiltrate of lymphocytes and macrophages was present in the superficial dermis. Colon and ileum biopsy results showed mucosal alterations with increased apoptotic activity and cellular regeneration at the crypt bases. Colonic biopsy revealed more severe lesions, with associated mucosal denudation, enlarged lamina propria, and diffuse lymphocytic infiltration. No hemophagocytosis was found in these tissues (see Figure 1A,B).

## Discussion

HLH is a rare disease characterized by uncontrolled proliferation of mature histiocytes, hemophagocytosis, and up-regulation of inflammatory cytokines. The more typical findings are fever, peripheral cytopenia affecting two lineages at least, hepatosplenomegaly, hypertriglyceridemia and/or hypofibrinogenemia, and hemophagocytosis. Recently, the Histiocyte Society updated its guidelines [1] and proposed to add three diagnostic criteria: hyperferritinemia, decreased or absent natural killer (NK) cell activity, and high soluble interleukin 2 receptor serum levels. Five among eight of these criteria should be fulfilled to confirm the diagnosis. They were present in our patient, who developed high persistent fever, severe pancytopenia, hepatomegaly on abdominal CT scan, and very high plasma levels of ferritin, triglycerides, and LDH.

Despite an extensive search, nasopharyngeal influenza A (H1N1) found on PCR was the only positive result able to explain the initial symptoms and secondary HLH. The clinical presentation of our patient was unusual, with predominantly digestive signs and no respiratory signs initially. Gastrointestinal symptoms are known to frequently occur in seasonal influenza and stool influenza A viral carriage has been recently documented in this situation [4]. A skin involvement may also be observed during influenza, with exanthema or petechial rash. In our patient, skin biopsy results were consistent with viral-induced lesions or a toxic epidermal necrosis. Ileum and colon histological features were more severe and consistent with a cell-mediated immune reaction or direct viral lesions. Previous studies have shown that severe apoptosis can be detected in the intestinal tissue of patients developing an influenza virus infection, with reactive hemophagocytic activity in bone marrow, lungs, and liver [5].

The first reported case of HLH associated with the H1N1 influenza virus was in a 17-year-old who completely recovered without immunosuppressive treatment [6]. Our patient's case was dramatically different, with rapid evolution to a lethal multi-organ failure syndrome.

The induction of HLH by H5N1 and H3N2 influenza virus has been previously described. These variants induce an ineffective cytotoxicity by CD8+ T lymphocytes, with a decreased perforin expression, both mechanisms potentially leading to HLH syndrome [1,7]. Whether the 2009 influenza A (H1N1) virus can bring on these changes is not yet known.

## Conclusions

Hemophagocytic lymphohistiocytosis with multi-organ failure syndrome can be induced by the novel H1N1 influenza variant. Despite its rarity, clinicians should be aware of this possibility to enable early recognition and treatment.

## Consent

Written informed consent was obtained from the patient's next-of-kin for publication of this case report and any accompanying images. A copy of the written consent is available for review by the Editor-in-Chief of this journal.

## Competing interests

The authors declare that they have no competing interests.

## Authors' contributions

CW analyzed the clinical and biological patient data and partially wrote the paper; AC and AW performed the histological examination and interpretation; MJG participated in patient data interpretation and writing of the manuscript; FF initiated the patient analysis and report, and wrote the final version of the manuscript. All authors read and approved the final manuscript.
